# Occurrence of Rare Pathogens at the Site of Periprosthetic Hip and Knee Joint Infections: A Retrospective, Single-Center Study

**DOI:** 10.3390/antibiotics10070882

**Published:** 2021-07-20

**Authors:** Konstantinos Anagnostakos, Christoph Grzega, Ismail Sahan, Udo Geipel, Sören L. Becker

**Affiliations:** 1 Zentrum für Orthopädie und Unfallchirurgie, Klinikum Saarbrücken, 66119 Saarbrücken, Germany; cgrzega@klinikum-saarbruecken.de (C.G.); ssahan@klinikum-saarbruecken.de (I.S.); 2 Bioscientia MVZ Saarbrücken GmbH, 66119 Saarbrücken, Germany; udo.geipel@bioscientia.de; 3 Institut für Medizinische Mikrobiologie und Hygiene, Universitätsklinikum des Saarlandes, 66421 Homburg/Saar, Germany; soeren.becker@uks.eu

**Keywords:** hip infection, knee infection, periprosthetic joint infection, antibiotic resistance

## Abstract

The frequency and clinical relevance of rare pathogens at the site of periprosthetic infections of the hip and knee joint and their antibiotic resistance profiles have not yet been assessed in-depth. We retrospectively analyzed all periprosthetic hip and knee joint infections that occurred between 2016 and 2020 in a single center in southwest Germany. Among 165 infections, 9.7% were caused by rare microorganisms such as *Veilonella* sp., *Pasteurella* sp., *Pantoea* sp., Citrobacter koseri, Serratia marcescens, Parvimonas micra, Clostridium difficile, Finegoldia magna, Morganella morganii, and yeasts. No resistance to piperacillin/tazobactam, carbapenemes, fluoroquinolones, or gentamicin was observed. Some bacteria displayed resistance to ampicillin, ampicillin/sulbactam, and cefuroxime. We present follow-up data of patients with infections due to rare pathogens and discuss the importance of close, interdisciplinary collaboration between orthopedic surgeons and clinical microbiologists to carefully select the most appropriate anti-infective treatment regimens for the increasing number of patients with such infections.

## 1. Introduction

Periprosthetic joint infections (PJI) after hip or knee arthroplasty are accepted to be a rare but hazardous complication with an overall incidence of 1–2% [1]. Infections significantly impact the clinical course of affected patients, as prolonged inpatient antibiotic therapy and repeated surgery are frequently required to effectively treat these conditions. The orthopedic community has increasingly acknowledged the importance of proper and timely diagnosis as well as adequate treatment of PJIs in recent years [2]. The causative pathogen is accordingly an important determinant of the clinical outcome. Indeed, it is known that multidrug-resistant organisms are associated with a poorer outcome and a higher risk of treatment failure [3,4].

Numerous studies have sought to investigate the exact epidemiology and microbiological etiology of PJIs in either cohort studies [1,5,6,7,8] or analyses of data from national registries [9,10,11]. All studies agree that staphylococci represent the most common causative organisms identified at the sites of PJIs, whereas some geographical differences might be observed [1]. For staphylococcal infections, there is compelling evidence regarding incidence, resistance patterns, and suggested treatment regimens [5,6,7,8,9,10,11]. In contrast, much less is known about other causative agents giving rise to PJIs, especially with regard to uncommon microorganisms that some studies summarized under the term “other pathogens” [5,6,7]. However, their exact identification, resistance profiles, and targeted treatments are certainly not of minor importance. Information about these rare organisms are currently available from either single reviews [12] or numerous case reports [13,14,15,16,17,18,19].

To the best of our knowledge, no study has dealt with this topic, yet. Hence, the aim of the present retrospective study was to describe the microbiological etiology of hip and knee PJIs in a large cohort at a single center over a 5-year period, with particular emphasis on rare pathogens that have been described infrequently as agents of PJIs in the peer-reviewed international literature.

## 2. Results

Between 2016 and 2020, 1654 arthroplasty surgeries of the hip and knee joint were performed in the department of the first author. Of that total, 1078 were primary surgeries (hip: 809, knee: 269), and 411 were carried out due to aseptic reasons (hip: 264; knee: 147).

In total, 165 cases of PJIs were documented during the study period. Of those, 100 infections affected hip prostheses, while 65 prosthetic infections of the knee were diagnosed. Fifty-six of the hip and fifty-seven of the knee patients, respectively, were not primarily operated on by our department, but were referred to us from other hospitals. There were more male than female patients and the mean age was 70.8 years (range: 35–89 years; Table 1).

Based on a combination of microbiological techniques, at least one microorganism was identified in 72.7% of the cases (120/165). There were 99 mono- and 21 polymicrobial infections. In 45 cases (27.2%), the results were negative (knee: 20/65; hip 25/100); 20 of the 45 negative cases received a pre-treatment with antibiotics (knee: 8/20; hip: 12/25).

Among the 120 cases with microbiological detection, 148 bacteria belonging to 34 different species could be identified. Gram-positive bacteria accounted for 80.4% (119/148) and Gram-negative pathogens for 17.6% (26/148) of the cases. Fungal infections were observed in 3 cases (2.0%). Staphylococci were the most common group of pathogens and were found in 54.7% of the cases. The distribution of all pathogens is displayed in Table 2.

Of these detected cases, 10.9% were already positive on the Gram stain (18/165). Of note, in one case, the staining was positive and correlated with positive histological findings (Type II). The Gram-negative rods seen in the staining of this particular case could not be cultivated, and a polymerase chain reaction (PCR) assays for bacteria was also negative. In five cases pathogens were exclusively identified by PCR, while microbiological cultures remained negative.

**Figure 1:**
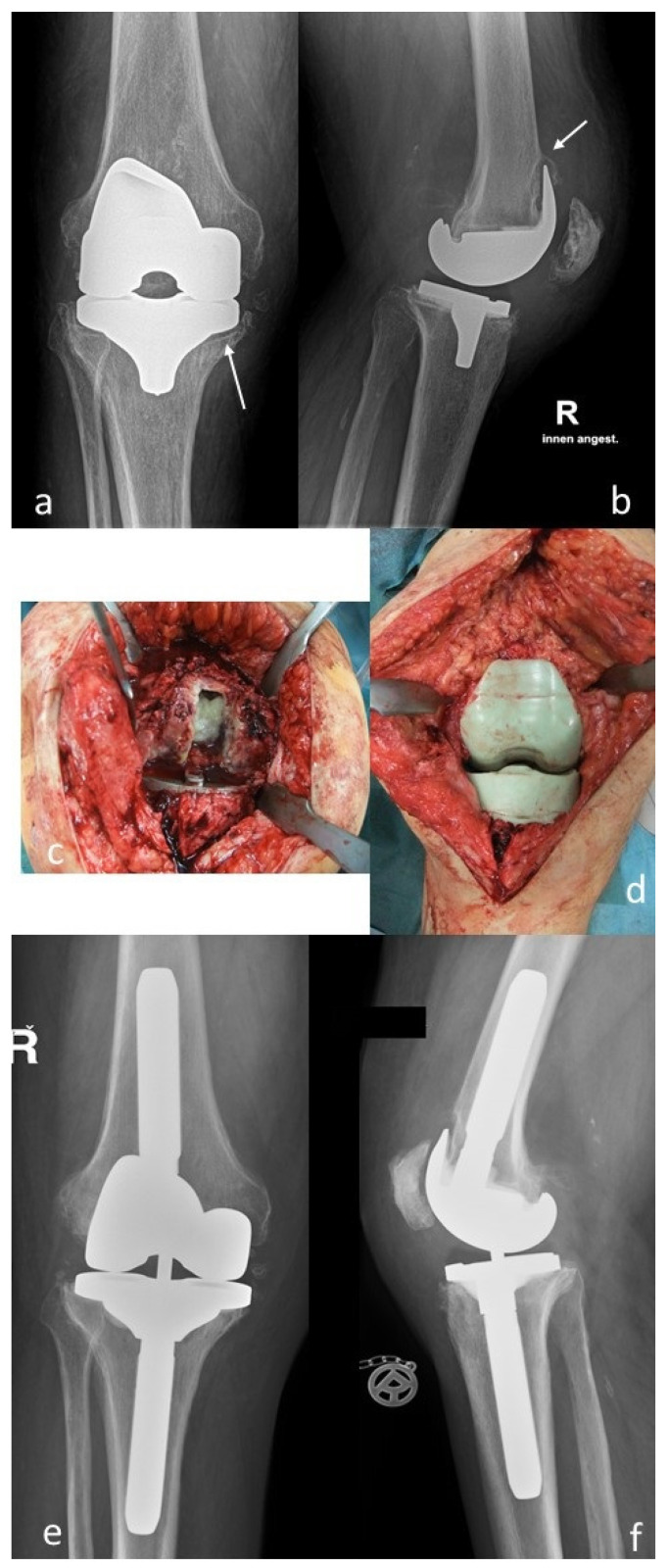
(**a**,**b**): Preoperative a.p. and lateral radiographs of the right knee joint of a 71-year-old male patient. Notice the osteolyses of the proximal medial tibia and the anterior part of the distal femur; (**c**,**d**): Intraoperative findings. After removal of the femoral component, pus was evident in the femoral canal (*Serratia marcescens*). An articulating antibiotic-loaded spacer was implanted for management of the infection; (**e**,**f**): After infection eradication, a condylar-constrained prosthesis was re-implanted.

**Figure 2:**
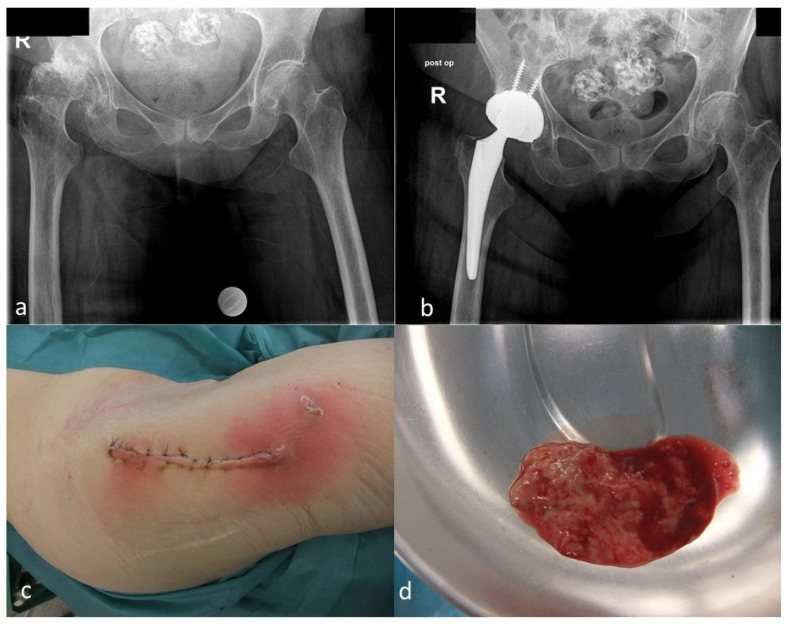
(**a**): Preoperative a.p. radiographs of the pelvis of a 79-year old female patient with a femoral head necrosis and secondary osteoarthritis of the right hip joint; (**b**): Postoperative radiographs after implantation of a cementless total hip arthroplasty; (**c**): Local findings 7 days after the surgery, indicating an early postoperative infection; (**d**): Purulent fluid was present in the joint (*Clostridum difficile*). The patient could be successfully treated by DAIR.

Based on our definition, a rare organism was observed in 16 cases (9.7%) (Table 2 and Table 4). There were 10 male and 6 female patients at a mean age of 68 (51–85) years. The comorbidities of these patients are presented in Table 3. The majority of the patients suffered from multiple comorbidities. There were 13 bacterial and 3 fungal infections. Primary surgical indications included primary total hip arthroplasty in eight cases, primary total knee arthroplasty in seven cases, and an aseptic acetabular cup revision arthroplasty in one case. DAIR procedures were carried out in seven cases and two-stage procedures in nine cases (Table 4) (Figure 1 and Figure 2).

The analysis of the resistance profiles of the identified bacteria (Table 4) did not show resistance to piperacillin/tazobactam, carbapenems, fluoroquinolones, or gentamicin. In some cases, bacterial strains were resistant to ampicillin, ampicillin/sulbactam, and cefuroxime. In four cases of anaerobic bacteria (*Clostridium difficile*, *Finegoldia magna*, *Parvimonas micra*, *Veillonella* spp.), no detailed resistance testing was carried out.

From the 16 patients, three were lost during follow-up and one passed away due to reasons not related to the PJI. Among the remaining 12 patients, three suffered from a reinfection with a causative organism different than the one primarily identified. The first patient had a reinfection with *Escherichia coli* (previously *Finegoldia magna*), the second one with methicillin-resistant *S. epidermidis* (primarily *Candida guilliermondii*), and the third one with *methicillin-susceptible Staphylococcus aureus* (primarily *Serratia marcescens*) (Table 5). All three underwent a two-stage procedure for further eradication of the infection.

## 3. Discussion

The aim of the present study was to determine the occurrence of rare microorganisms at the site of hip and knee PJIs and evaluate their antibiotic resistance patterns at a single center. Our results demonstrate that such uncommon pathogens accounted for 9.7% of all cases. None of these organisms was multi-drug resistant. However, in 25% of the cases, that were followed up, reinfections occurred with organisms other than those primarily identified.

The microbiological spectrum at the sites of PJIs, and in some cases their resistance profile, has been described in various studies. In a retrospective single-center study, Rafiq et al. found that coagulase-negative staphylococci were the most common organism in 67% of the cases in a cohort of 337 infected THAs [7]. “Other” organisms were responsible for 5% of the cases. Similar findings were described by Nickinson et al. at the sites of 121 infected knee arthroplasties [6]. Coagulase-negative staphylococci were the dominant group in 49% of the cases, whereas “other” organisms were seen in 25% of the cases. Drago et al. evaluated the microbiological findings of 429 PJIs of the hip and knee [5]. Staphylococci were the most frequent organism in 66.6% of the cases, followed by Enterobacteriaceae and *Cutibacterium acnes* [7]. There were no differences in the findings between hip and knee PJIs. Among the “rarely” identified organisms, *Acinetobacter* sp. were observed in 4 cases, *Corynebacterium* species in 10, *Candida* species in 1, and other anaerobes in 9 infections. In a very detailed retrospective study of 294 hip and knee PJI cases, Tsai et al. reported that the most common pathogenic organism was methicillin-susceptible *S. aureus* (26.5%), followed by coagulase-negative *staphylococci* (14.3%) [1]. Culture-negative findings were present in 27.2% of the cases. A variety of rare organisms, such as *Prevotella* species, *Parvimonas micra*, *Salmonella enterica*, and *Morganella morganii* could be identified. Interestingly, fungal and *Mycobacterium* infections were observed in 1.7% of the cases.

To the best of our knowledge, the term “rare” has not yet been unambiguously defined in the literature with regard to orthopedic infections. Several terms, such as “rare”, “atypical”, and “unusual”, have been used for the description of organisms that are not frequently identified at the sites of PJIs [12,18,20,21]. Caution must be exercised when trying to propose such a definition because rigorous scientific and clinical criteria are lacking, and such criteria might vary among different medical disciplines. The present definition used here sought solely to identify the “true” rare organisms; however, we cannot disregard the fact that under other circumstances (geographical differences, larger/smaller cohorts, etc.) the rate of rare organisms might differ from the one identified in the present study. Especially the geographical differences are of great importance. Aggarwal et al. evaluated all PJIs treated over a period of 12 years at two referral centers: one in Europe and the other in the United States [22]. The incidence of methicillin-resistant staphylococcal species, and particularly *S. aureus*, was significantly higher in the US than in Europe. Likewise, 27% of the *Enterococcus* infections were vancomycin-resistant in the US, whereas no isolates in Europe showed such resistance.

There are various possible causes for the increasing detection of rare organisms. First of all, the number of revision arthroplasty surgeries is increasing worldwide. Even if the particular revision rates stay the same, the absolute numbers will increase, and thus the possibility of identifying more pathogenic organisms. Over the past 10–15 years, a significant number of new bacterial species or subgroups within known species has been described [23]. The cases that were previously just called, for example, “staphylococci with no further differentiation” can nowadays be classified into numerous subgroups [23]. Furthermore, new microbiological detection methods have been developed and established in clinical practice. At the sites of implant infections, the use of sonication is recognized to have additional advantages with regard to sensitivity and specificity compared to the gold standard bacterial cultures [24,25]. The use of molecular biological techniques, such as polymerase chain reaction (PCR), also count as an enhancement to diagnostic measures despite their susceptibility to contamination and inhibition [26,27,28]. Prolonged cultivation periods in the range of ≥ 14 days compared with standard cultures over 7 days demonstrated an increase in the detection rate by more than 25% [29], although more recent studies question the necessity of extended culture duration in acute periprosthetic hip and knee joint infections [30]. Last but not least, more tissue samples are nowadays taken during surgery and sent for further microbiological examination, thus increasing the possibility of a positive microbiological result. The Infectious Diseases Society of America (IDSA) recommends submitting at least three and optimally five or six periprosthetic tissue samples for aerobic and anaerobic culture [31].

Regarding the results presented here, it is important not to over-interpret the relative frequency of each identified organism. Such values are dynamic and greatly depend on “trends” of diagnostics and treatment. The more revision surgeries are performed, the more tissue samples are taken and investigated, and with the improvement of diagnostic measures, the higher is the possibility of identifying more pathogenic agents. Our study acknowledges the increasing relevance of these lesser known pathogens with regard to musculoskeletal infections and in particular PJIs. The origin of infections caused by these pathogens frequently remains unknown, but might have been hematogenous in some cases. Indeed, many of these bacteria belong to the physiological microbiota environment in other parts of the human body, as is exemplarily shown in Table 6 for some of the pathogens detected in our study.

Data in the literature on PJIs are scarce for the rare pathogens found in our study. Some of these organisms (*P. micra*) have been described at the sites of PJIs following dental procedures [32], although there is considerable debate as to whether an antibiotic treatment should be provided in the prevention of those infections [33]. Others, such as *F. magna*, have been seen either at the site of polymicrobial infections [34] or in a single report of two cases [13]. PJIs due to *Veilonella* species [14,16,35], *Pantoea* species [19], *Pasteurella* species [15,36,37,38,39,40,41], and *Citrobacter* [42,43] are exceedingly rare. Of note, most of these bacteria (9/13) are Gram-negative. It is generally accepted that the eradication of Gram-negative PJIs can be difficult, with success rates ranging between 52 and 75%, depending on whether DAIR or two-stage procedures have been carried out [44,45]. Similar results have been reported for fungal PJIs. At the sites in 31 cases, Azzam et al. reported that 70% of the patients treated with DAIR suffered from infection persistence and required resection arthroplasty [46]. However, only 9 of the 29 patients undergoing resection arthroplasty underwent eventual eradication of the infection and delayed reconstruction. In a systematic review of surgical treatments (one-stage, two-stage, resection arthroplasty, DAIR) and clinical outcomes, Fusini et al. observed total success in 63% of the cases [47]. Kuiper et al. described an 85% success rate when two-stage exchange arthroplasty was performed [48]. Overall, it is apparent that all these scarce reports with a discrepancy of outcomes do not allow for a generalization of conclusions.

The usual perioperative antibiotic prophylaxis involves a first- or second-generation cephalosporin (cefazolin or cefuroxime) regardless of the type of surgery (primary or revision) or comorbidities of the patient [49]. This choice is often appropriate because Gram-positive bacteria are responsible for the majority of PJIs [5]. On the other hand, difficult-to-treat PJIs are becoming an increasing problem [50,51], and such antibiotic therapy might not be effective against these infections. The resistance patterns of the organisms in the present study show that these organisms were not multi-drug resistant and were susceptible to a wide range of tested antibiotics in vitro. In single cases, resistance was seen against ampicillin and cefuroxime, mainly in *Serratia marcescens*, which is intrinsically resistant to ampicillin. No resistance was observed against fluoroquinolones, and especially ciprofloxacin, which is regarded to be a cornerstone in the treatment of Gram-negative PJI [52]. Despite the antibiotic susceptibility of these organisms, 25% of the patients that were followed up suffered from reinfections with an organism other than primarily identified. We do not regard this as a failure of treatment. It is generally accepted that successful treatment of PJI does not depend solely on systemic antibiotic therapy, but also on other factors such as surgical debridement, local antibiotic therapy, or patient comorbidities. In particular, the presence of certain comorbidities such as diabetes mellitus, obesity, hypertension, hepatitis C, drug abuse, and heart and renal disorders are recognized to be risk factors for the emergence of PJI in general [1,8]. The sole role of each comorbidity in the emergence of a PJI caused by a rare organism is, however, unclear, and difficult to evaluate based on the small number of patients identified in the present work as well as the limited data in the literature.

Several limitations of our study are presented for consideration. The study was retrospective, with all the drawbacks of such a design. Due to this design, we were not able to determine which antibiotics were previously received by patients who were referred to us. The presently suggested definition of “rare organisms” is a first attempt, and might be further modified in the future. The 16 cases evaluated did not allow for a generalization of conclusions about the pathogenicity of these organisms at the sites of PJIs. Finally, further progress in infectious disease diagnostics will certainly change and improve our understanding of the microbiological etiology of PJIs in the foreseeable future, e.g., by the introduction of metagenomic sequencing in routine clinical practice [53].

## 4. Materials and Methods

A retrospective analysis of the internal arthroplasty data bank of the department of the first author was performed for identification of all periprosthetic hip and knee joint infections during the time period 2016–2020. Inclusion criteria were all revisions that were performed due to septic reasons, with complete documentation of all diagnostic measures. Patients that had revision arthroplasty surgery for any other reasons, and those with insufficient or incomplete documentation, were excluded from the study. Due to the retrospective study design, approval by the local ethics committee was unnecessary.

The primary aim of the study was to identify the rates and resistance patterns of rare pathogenic organisms at the sites of hip and knee PJIs. The secondary goal was to determine infection eradication rates at the sites of these rare infections.

Infections included in this analysis were defined by the criteria of the Musculoskeletal Infection Society (MSIS) [54]. Preoperatively, a joint aspiration was performed to differentiate aseptic from septic prosthesis loosening, except for those patients whose positive blood cultures confirmed hematogenous infections or who presented with systemic sepsis signs and were immediately operated on. A further exclusion concerned patients who had fistulas. In these cases, we preferred to take direct tissue samples during surgery. If joint aspiration revealed negative microbiological findings, but clinical, laboratory or radiological findings pointed strongly to the presence of an infection, an arthroscopic or open biopsy was performed prior to the prosthesis revision.

### 4.1. Surgical Management

All patients, suffering from an early or acute hematogenous PJI were initially treated by means of DAIR (debridement, antibiotics, irrigation, retention (of prosthesis)). All infected, necrotic, or ischemic tissue layers were debrided. Removable prosthetic components (knee: polyethylene insert; hip: acetabular cup insert, femoral head) were always exchanged. A pulsatile lavage with at least 5 L Ringer’s solution was also performed.

All patients with a late PJI and those having had two unsuccessful DAIR surgeries with persistence of infection [55] underwent a two-stage procedure. In the first surgery, all prosthetic components including cement were removed, and all infected, necrotic, or ischemic tissue layers were debrided. A pulsatile lavage with at least 5 L Ringer’s solution was always performed.

At the sites of hip infections, the primary goal has always been to implant an antibiotic-loaded spacer. In these cases, the spacer was intraoperatively produced by means of commercially available molds (Stage One^TM^, Fa. ZimmerBiomet, Freiburg im Breisgau, Germany). However, patients with a reduced medical condition and unable to avoid putting any weight on the operated extremity postoperatively, those who suffered from large osseous defects of the proximal femur or acetabulum, and those who needed a transfemoral approach for the safe removal of the femoral stem were deemed better suited for a resection arthroplasty (Girdlestone procedure) due to the higher theoretical risk of a secondary spacer dislocation or fracture during the interim phase [56]. In these cases, 2–3 antibiotic-loaded beads (Septopal^®^, Fa. ZimmerBiomet, Freiburg im Breisgau, Germany) were inserted into the acetabulum and the femoral canal.

Regarding knee infections, the presence of bone defects, according to the Anderson Orthopedic Research Institute (AORI) bone defect protocol [57], helped us decide whether an articulating or a static spacer should be implanted. All patients with bone defects I-IIA were treated with an articulating spacer (Copal knee molds, Fa. Hereaus, Wehrheim, Germany). Patients suffering from bone defects IIB-III were treated with a static spacer. This spacer was molded individually according to the particular joint space geometry.

For the intraoperative production of hip and knee spacers, commercially available antibiotic-loaded bone cement was used, loaded either with gentamicin or gentamicin + clindamycin (Palacos^®^ R + G/Copal^®^ G + C, Fa. Hereaus, Wehrheim, Germany). Depending on the particular causative organism and its resistance profile, 2 g vancomycin/40 g bone cement were additionally incorporated into the cement in certain cases.

After the operation, an immediate, systemic antibiotic therapy was started—either specific if the causative organism was preoperatively known, or a calculated therapy with 1.5 g cefuroxime intravenously (thrice daily) if the causative organism were unknown, and adjusted if necessary during the further course. All patients received an antibiotic therapy over 6 weeks, consisting of administration 3–4 weeks intravenously and 2–3 weeks orally. All knee joints with a static spacer were immobilized in a cast in full extension. Patients with an articulating spacer were allowed to flex their knee as tolerated. All patients (hip and knee) were allowed to walk on crutches with no weight on the operated extremity.

Six weeks after the spacer implantation or the Girdlestone procedure, the antibiotic therapy was paused for 7–10 days and the serum inflammation parameters (C-reactive protein, blood cell count) controlled. If the laboratory parameters were normal, a prosthesis reimplantation was then planned if the wound had healed and the general medical condition of the patient allowed for it. The types of implants used were chosen based on the amount of bone loss and quality. A joint aspiration was not routinely carried out prior to spacer explantation and prosthesis reimplantation because data in the literature demonstrated no benefit from such a measure [58,59].

### 4.2. Microbiological and Histopathological Diagnostic Techniques

Tissue samples from at least 5 different locations along with joint fluid (when present) were taken and sent for further microbiological and histological examination. All samples were sent within 30 min to our Microbiologic and Pathologic Institute.

Upon histopathological analysis, all samples were classified in accordance with the system of Krenn and Morawietz to provide an estimate of the probability of infection [60].

Upon receipt for microbiological analysis, all samples were immediately processed and subjected to microscopic examination using a Gram stain. Different agar media and enrichment broths were used for microbiological culture, i.e., Columbia blood agar and MacConkey agar for aerobic culture, and Schaedler agar plates for anaerobic culture. Thioglycolate bouillons of each sample were inspected daily for an incubation period of 7 days until 2018, which was then prolonged to 14 days per sample to account for any slowly growing bacteria [29]. Culture-grown colonies of bacteria or fungi were subjected to further analysis using matrix-assisted laser desorption/ionization time-of-flight (MALDI-TOF) mass spectrometry or the VITEK^®^ 2 system (BioMérieux; Nürtingen, Germany) for species identification. Antimicrobial susceptibility testing was performed using either the VITEK^®^ 2 system or agar disc diffusion tests, as appropriate. Samples without microbiological growth but with suspicious histopathology results were further analyzed by a broad-range 16S rRNA PCR to assess the presence of bacterial nucleic acids. PCR-positive samples were subsequently sequenced to reach identification at the genus or species level.

For the organisms exclusively identified by 16S PCR, no resistance testing could be performed. All laboratory procedures were in accordance with the microbiological-infectiological quality standards (MiQs) of the German Society of Hygiene and Microbiology.

### 4.3. Definition of Rare Organisms

The term “rare” has not been unambiguously defined with regard to PJI. Therefore, we had to subjectively set classification criteria. This decision was made by both the treating orthopedic surgeons and the microbiologists involved in the management of these cases.

First of all, we decided not to define the rarity of the organisms solely based on their occurrence in our study, to avoid setting-specificity. Moreover, we sought to distinguish between the truly rare organisms and the “unusual” or “atypical” ones. The latter organisms would probably be those that are frequently identified at the sites of other infections (e.g., pneumonia, urinary tract infections), but not typically at the site of a PJI. In our opinion, however, they do not warrant automatic classification as “rare” and rather should be named “unusual” or “atypical”. Last but not least, we searched the international peer-reviewed literature in English for every pathogenic organism detected in the present study in order to identify whether there were many or only a few scientific publications about that particular organism.

Following these preparatory steps, an organism was defined as “rare” if (1) it was not typically associated with PJI, or (2) it had only been described in a maximum of 10 case reports or a small case series about PJIs in the English literature. An exclusion applied here to fungal infections, because these are the only organisms that are recognized to be rare causative organisms in PJIs according to the literature, with an incidence rate of less than 1% [46,47,48].

## 5. Conclusions

To our knowledge, the present study is the first that sought to determine the occurrence of rare organisms and their antibiotic resistance patterns at the sites of hip and knee PJIs. Such pathogens accounted for approximately 10% of all infections. No organism was multi-drug resistant or difficult to treat. Although orthopedic surgeons are responsible for the practical treatment, these findings call for the establishment of close, interdisciplinary collaboration with clinical microbiologists and infectious disease specialists to carefully select the most appropriate anti-infective treatment options for patients suffering from PJIs due to such less-common causative agents.

## Figures and Tables

**Table 1:** Demographic data in a study on the microbiological etiology of hip and knee prosthetic joint infections at a single center in southwest Germany, 2016–2020.

Treatment Category	*n* =	Gender	Mean Age (y.) (Min–Max)
Total cohort	165	76 f/89 m	70.8 (35–89)
Hip—total	100	52 f/48 m	72 (35–89)
hip—DAIR	49	22 f/29 m	71.9 (35–89)
hip—2-stage *	51	30 f/19 m	72.1 (35–89)
knee—total	65	24 f/41 m	69.1 (51–87)
knee—DAIR	12	5 f/7 m	69.9 (57–80)
knee—2-stage	53	19 f/34 m	68.9 (51–87)

DAIR: debridement, antibiotics, irrigation, retention (of prosthesis); f: female; m: male; *: 12× spacer implantation, 37× Girdlestone hip; y.: years.

**Table 2:** Overview of the identified 148 organisms at the sites of 165 periprosthetic hip and knee joint infections.

Microorganism	*n* (Percentage)
*Staphylococcus epidermidis*	43 (29.1%)
Methicillin-resistant *S. epidermidis* (MRSE)	33 (22.3%)
Methicillin-susceptible *S. epidermidis* (MSSE)	10 (6.8%)
*Staphylococcus aureus*	26 (17.6%)
Methicillin-resistant *S. aureus* (MRSA)	2 (1.4%)
Methicillin-susceptible *S. aureus* (MSSA)	24 (16.2%)
*Enterococcus faecalis*	13 (8.8%)
Beta-hemolytic *streptococci*	9 (6.0%)
*Escherichia coli*	6 (4.0%)
*Serratia marcescens*	4 (2.7%)
*Pseudomonas aeruginosa*	4 (2.7%)
*Enterococcus faecium*	4 (2.7%)
*Staphylococcus caprae*	3 (2.0%)
*Staphylococcus haemolyticus*	3 (2.0%)
*Enterobacter cloacae*	3 (2.0%)
*Staphylococcus hominis*	3 (2.0%)
*Cutibacterium acnes*	3 (2.0%)
*Staphylococcus warneri*	2 (1.3%)
*Streptococcus gallolyticus*	2 (1.3%)
*Parvimonas micra*	2 (1.3%)
*Candida albicans*	2 (1.3%)
*Citrobacter koseri/diversus*	1 (0.6%)
*Pasteurella sp.*	1 (0.6%)
*Proteus mirabilis*	1 (0.6%)
Alpha-hemolytic *streptococci* (not further specified)	1 (0.6%)
*Klebsiella pneumoniae*	1 (0.6%)
*Staphylococcus capitis*	1 (0.6%)
*Pantoea* sp.	1 (0.6%)
*Clostridium difficile*	1 (0.6%)
*Finegoldia magna*	1 (0.6%)
*Streptococcus oralis*	1 (0.6%)
*Enterobacterales* (not further specified)	1 (0.6%)
*Streptococci*–(not further specified)	1 (0.6%)
*Veilonella parvula/ tobetsuensis*	1 (0.6%)
*Candida guilliermondii*	1 (0.6%)
*Morganella morganii*	1 (0.6%)

n: absolute number; sp.: species.

**Table 3:** Baseline characteristics and comorbidities of the 16 patients suffering from PJI with rare organisms.

Patient	Gender	Age	Comorbidities
1	f	52	NIDDM, chronic venous insufficiency, hypothyreosis, hepatitis C, drugs abuse
2	m	51	depression
3	f	64	arterial hypertension, obesity
4	f	79	renal insufficiency, heart insufficiency, peripheral arterial obstructive disease, cerebral hemorrhage, atrial fibrillation
5	m	71	arterial hypertension, obstructive sleep apnea syndrome, NIDDM, coronary heart disease with stents implantation, gout, colon cancer
6	m	77	none
7	m	56	splenectomy due to hereditary spherocytosis
8	m	56	NIDDM, coronary heart disease with bypass surgery, anxiety disorder
9	f	82	arterial hypertension, Alzheimer’s disease
10	m	71	arterial hypertension, atrial fibrillation, anxiety disorder
11	m	68	none
12	m	69	none
13	m	85	arterial hypertension, coronary heart disease, atrial fibrillation
14	f	67	renal insufficiency, atrial fibrillation, Ogilvie syndrome
15	m	79	pulmonary hypertension, heart insufficiency,
16	f	63	arterial hypertension, osteoporosis, obesity, stomach stapling operation

**Table 4:** Overview of rare bacteria and their antibiotic resistance profiles, detected in a study on the microbiological etiology of hip and knee prosthetic joint infections at a single center in southwest Germany, 2016–2020.

Causative Bacterium	*M. morganii*	*V. parvula/tobetsuensis* *	*F.* *magna*	*Cl. difficile*	*Pantoea* sp.	*Pasteurella* sp.	*P.* *micra* (1)	*P.* *micra* (2) *	*C.* *koseri/diversus*	*S. marcescens* (1)	*S. marcescens* (2)	*S. marcescens* (3)	*S. marcescens* (4)
Ampicillin	r	n.t.	n.t.	n.t.	s	s	s	n.t	r	i	r	r	i
Ampicillin/sulbactam	i	n.t.	n.t.	n.t.	s	s	s	n.t	s	s	i	i	s
Piperacillin	s	n.t.	n.t.	n.t.	s	s	s	n.t	i	s	s	s	s
Piperacillin/tazobactam	s	n.t.	n.t.	n.t.	s	s	s	n.t.	s	s	s	s	s
Cefuroxime	s	n.t.	n.t.	n.t.	s	s	s	n.t.	s	r	r	r	r
Cefpodoxime	s	n.t.	n.t.	n.t.	s	s	s	n.t.	s	s	s	s	s
Cefotaxime	s	n.t.	n.t.	n.t.	s	s	s	n.t.	s	s	s	s	s
Ceftazidime	s	n.t.	n.t.	n.t.	s	i	s	n.t.	s	s	s	s	s
Imipenem	s	n.t.	n.t.	n.t.	s	s	s	n.t.	s	s	s	s	s
Meropenem	s	n.t.	n.t.	n.t.	s	s	s	n.t.	s	s	s	s	s
Ertapenem	s	n.t.	n.t.	n.t.	s	n.t.	s	n.t.	s	s	s	s	s
Ciprofloxacin	s	n.t.	n.t.	n.t.	s	s	s	n.t.	s	s	s	s	s
Moxifloxacin	s	n.t.	n.t.	n.t.	s	s	s	n.t.	s	s	s	s	s
Gentamicin	s	n.t.	n.t.	n.t.	s	s	s	n.t.	s	s	s	s	s
Tigecycline	r	n.t.	n.t.	n.t.	s	n.t.	s	n.t.	s	i	s	s	s
Co-trimoxazole	s	n.t.	n.t.	n.t.	s	s	s	n.t.	s	s	s	s	s

S: susceptible; i: intermediate susceptible; r: resistant; n.t.: not tested; *: identification through 16S-rRNA PCR. (1), (2), (3), (4): means that this bacterium was detected in 4 different clinical cases; each number represents one case.

**Table 5:** Data on surgical and systemic antibiotic treatments and infections in a study on the microbiological etiology of hip and knee prosthetic joint infections at a single center in southwest Germany, 2016–2020.

Organism	Joint	Primary Surgical Indication	Treatment Procedure	Systemic Antibiotic Therapy	Follow-Up (Months)	Infection Eradication
*Morganella morganii* * (+ *MSSA*, *E. faecalis*)	hip	primary THA	two-stage	rifampicin + vancomycin	lost	unclear
*Veilonella parvula/tobetsuensis*	knee	primary TKA	two-stage	levofloxacine	12	yes
*Finegoldia magna*	hip	primary THA	DAIR	ciprofloxacine	7	no
*Clostridium difficile*	hip	primary THA	DAIR	rifampicin + ceftriaxone	10	yes
*Pantoea* sp.	knee	primary TKA	DAIR	rifampicin + ciprofloxacin	13	yes
*Pasteurella* sp.	knee	primary TKA	DAIR	rifampicin + cefuroxime/ciprofloxacine	54	yes
*Parvimonas micra*	hip	primary THA	two-stage	moxifloxacine	42	yes
*Parvimonas micra*	hip	primary THA	two-stage	ciprofloxacine	8	yes
*Citrobacter koseri/diversus*	hip	primary THA	two-stage	meropenem + ciprofloxacine	lost	unclear
*Serratia marcescens*	knee	primary TKA	two-stage	ciprofloxacine	58	yes
*Serratia marcescens*	hip	primary THA	DAIR	rifampicin + meropenem/ ciprofloxacine	34	yes
*Serratia marcescens*	hip	acetabular cup revision	DAIR	rifampicin + meropenem/ ciprofloxacine	36	yes
*Serratia marcescens*	knee	primary TKA	two-stage	ciprofloxacine	6	no
*Candida albicans* * (+ *E. coli*, *E. faecium*)	knee	primary TKA	two-stage	meropenem + teicoplanin + fluconazole	lost	unclear
*Candida albicans*	hip	primary THA	DAIR	fluconazole	exitus	n.r.
*Candida guilliermondii*	knee	primary TKA	two-stage	voriconazole	19	no

THA: total hip arthroplasty; TKA: total knee arthroplasty; DAIR: debridement, antibiotics, irrigation, retention of prosthesis; *: polymicrobial infection; n.r.: not relevant.

**Table 6:** Microbiological information about the rare causative bacteria.

Bacterium	Gram Stain	Aerobic/ Anaerobic	Family	Microscopic Morphology	Physiologic Environment
*Morganella morganii*	negative	facultatively anaerobic	Morganellaceae	rods	normal flora in intestinal tracts of humans, mammals, and reptiles
*Veilonella* sp.	negative	anaerobic	Veilonellaceae	cocci	normal flora in intestinal tracts and oral mucosa from mammals
*Finegoldia magna*	positive	anaerobic	Clostridia	cocci	normal flora on human skin, mucous membranes
*Clostridium difficile*	positive	anaerobic	Clostridioides	rods	normal flora in intestinal tracts of humans
*Pantoea* sp.	negative	facultatively anaerobic	Erwiniaceae	rods	plant surfaces, seeds, fruit, animal/human feces
*Pasteurella* sp.	negative	facultatively anaerobic	Pasteurellaceae	rods	oral flora from cats and dogs
*Parvimonas micra*	positive	anaerobic	Peptoniphilaceae	cocci	oral flora in humans
*Citrobacter koseri/diversus*	negative	facultatively anaerobic	Enterobacteriaeae	rods	normal flora from human and animal digestive tracts
*Serratia marcescens*	negative	facultatively anaerobic	Yersiniaceae	rods	human and animal digestive tracts, dust, soil, surface waters

## Data Availability

The data presented in this study are available on request from the corresponding author.
